# Pediatric Myofibroma of the Palate with Ulceration and Bone Destruction

**DOI:** 10.1155/2016/1432764

**Published:** 2016-06-14

**Authors:** Joseph A. Capo, Dina Moubayed, Sami P. Moubayed, Juan C. Hernandez-Prera, Azita Khorsandi, Daniel Buchbinder, Mark L. Urken

**Affiliations:** ^1^Department of Otolaryngology-Head and Neck Surgery, Mount Sinai Beth Israel, New York, NY 10003, USA; ^2^Faculty of Medicine, University of Montreal, Montreal, QC, Canada H3T 1J4; ^3^Department of Pathology, Mount Sinai Beth Israel, New York, NY 10003, USA; ^4^Department of Radiology, Mount Sinai Beth Israel, New York, NY 10003, USA

## Abstract

Myofibroma is a rare benign neoplasm occurring in the head and neck, arising primarily in infants and children. Frequently, myofibromas grow rapidly leading to suspicion of malignancy and the potential for overaggressive surgical excision. We aim to report a rare case of myofibroma with ulceration and bone destruction. A nine-year-old female presented with an ulcerated left hard palate mass. Open biopsy was performed with pathology suggestive of myofibroma. A left partial maxillectomy and reconstruction with a buccal advancement flap were performed. Final pathology confirmed the diagnosis of a benign myofibroma. Myofibroma is a rare benign tumor of the head and neck which must be considered in the differential diagnosis by the clinician and the pathologist in order to prevent inappropriate and/or overaggressive treatment.

## 1. Introduction

In the pediatric patient, the differential diagnosis of a mass on the hard palate includes inflammatory, congenital, and neoplastic conditions, in descending frequency [1]. Specific diagnoses include odontogenic infections, mucoceles, nasopalatine duct cyst, and minor salivary gland tumors [1]. A lesion in this area is typically initially investigated with imaging if deemed necessary and fine needle aspiration or open biopsy when the diagnosis is still uncertain. When rapidly growing, suspicion for malignancy increases.

We present a case of a rapidly enlarging myofibroma of the hard palate which initially raised concern for malignancy due to bone destruction. Myofibroma is a rare benign neoplasm occurring in the head and neck, arising primarily in infants and children [2]. We emphasize the importance of accurate preoperative diagnosis using imaging and pathology to limit over-aggressive surgical management. This is only the second reported case of myofibroma of the palate associated with bone destruction.

## 2. Case Report

**Figure 1:**
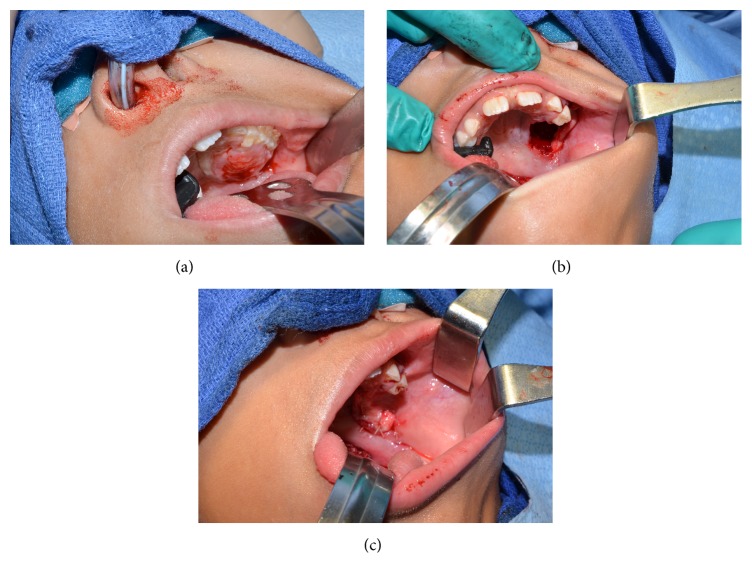
Physical examination showing an ulcerated mass on the left hard palate (a), the status of the palatal defect after resection (b), and the reconstructing using mucosal advancement flap (c).

**Figure 2:**
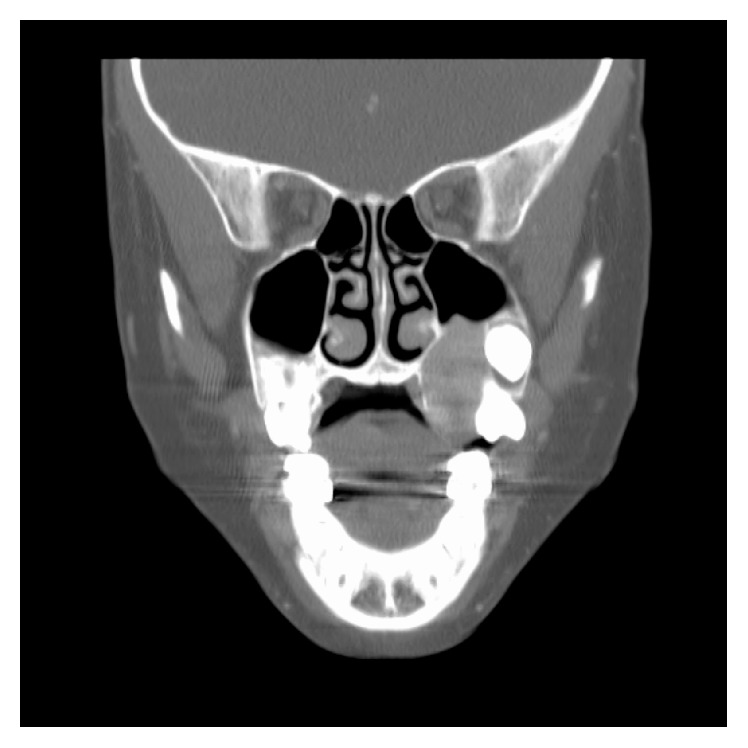
CT scan of the maxillofacial region in coronal view showing a destructive lesion of the left hard palate growing into the maxillary sinus.

A nine-year-old female was referred to our department for a mass arising in the left hard palate (Figure 1(a)) that had been reportedly growing for the past several weeks and was associated with discomfort with mastication and teeth brushing, as well as minor bleeding. Examination showed an ulcerated firm mass of the left hard palate. The ulcer was not opposing the inferior dentition. CT scan showed a 2.2 × 1.3 × 2.6 cm heterogeneously enhancing expansile mass of the left lateral hard palate with bone destruction, extending into the alveolar recess and maxillary antrum. There was no evidence of orbital extension (Figure 2).

A first biopsy was performed at an outside center and reviewed at out institution. The biopsy consisted of a small fragment of ulcerated squamous mucosa with a submucosal bland spindle cell proliferation. Due to the limited biopsy and lack of material to perform further immunohistochemical analysis, it was not possible to establish the nature of the tumor. Consequently, an open biopsy was performed and showed that the spindle cells were characterized by elongated cigar-shaped nuclei, eosinophilic cytoplasm with indistinct borders, and a storiform to fascicular growth pattern. Significant nuclear pleomorphism, increased mitotic activity, and necrosis were absent. The spindle cells showed variable immunoreactivity for muscle specific and smooth muscle actin and had a low Ki67 proliferation index (<5%); they were negative for desmin, S100 protein, cytokeratin (AE1/AE3), and nuclear beta-catenin. The overall findings supported the diagnosis of a myofibroma.

**Figure 3:**
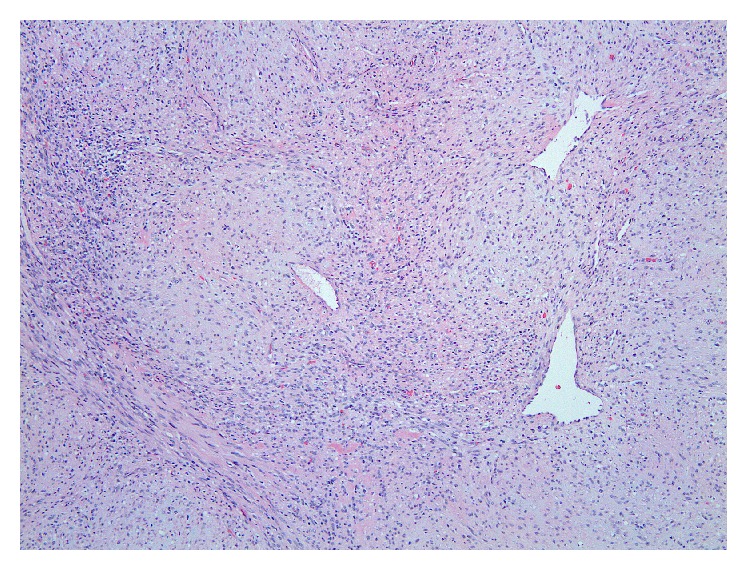
Bland spindle cell proliferation with nodular appearance and focal hemangiopericytoma-like vasculature (hematoxylin-eosin stain, original magnification ×100).

**Figure 4:**
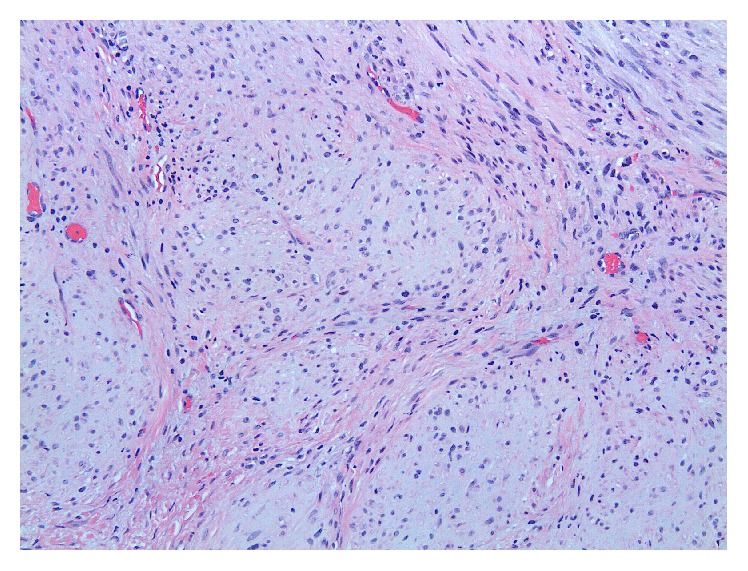
Fascicles of elongated spindle cells admixed with whorls of plumper pale-staining spindle cells (hematoxylin-eosin stain, original magnification ×200).

**Figure 5:**
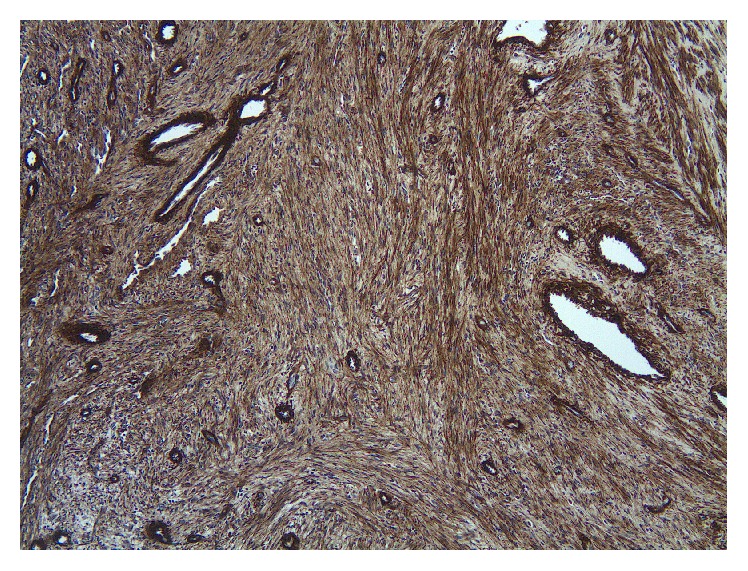
Tumor cells show shows strong reactivity for muscle specific actin (HHF35) (immunohistochemical stained-section, original magnification ×200).

The patient was brought back to the operating room for resection which involved a left partial maxillectomy and reconstruction with a buccal advancement flap (Figures 1(b) and 1(c)). The tumor was macroscopically completely resected despite a positive deep margin. The bone of the remaining tumor bed was drilled over the entire surface, which ensured elimination of the microscopically positive deep margin. Postoperatively, the patient tolerated a full liquid diet and was discharged the next day. Final pathology confirmed the diagnosis of a benign myofibroma. The microscopic examination of the resected specimen showed a submucosal encapsulated but well-circumscribed lesion with focal hemangiopericytoma-like vasculature and a nodular low magnification appearance (Figure 3). The latter pattern was secondary to fascicles of elongated spindle cells admixed with whorls of plumper pale-staining spindle cells (Figure 4). Smooth muscle differentiation was once more confirmed by immunohistochemistry (Figure 5). At 6-month follow-up, the patient remains free of disease.

## 3. Discussion

Myofibroma is a disease of the young, developing mainly in the first 2 years of life, but can occur in patients up to 40 years of age [2]. Previous reports have sited the mandible as the most common oral cavity site of myofibroma followed by the tongue, buccal mucosa, palate, and lip [2].

When compared to the previously reported patients [2], our patient falls into the reported age distribution (6–27). Most cases have reported lesional growth over a period of 1.5 to 2 months, similar to our patient. The majority (4/7) of previously reported cases were male, contrary to our patient. Finally, a majority of the reported cases of palatal myofibroma (5/7) did not show ulcer formation. Only one other case showed bone resorption.

Diagnosis is difficult on the basis of clinical, radiographic, and pathological features. Ulceration is a tumor characteristic that may be suggestive of a more aggressive biologic potential and can increase the clinician's suspicion for malignancy. In one review of the literature, of the 11 ulcerated tumors, 6 (54%) grew rapidly compared to just 4 (5%) of the 78 nonulcerated oral myofibromas [2]. Radiologically, up to 27.7% of palatal myofibroma tumors may show bone destruction, which also increases suspicion of malignancy [2], although only one case of bone destruction in the hard palate has been described previously [2]. Bone destruction in this benign nonosseous tumor remains pathophysiologically unclear.

Microscopic evaluation of the lesion with a proper use of immunohistochemical stains leads to the correct diagnosis. Myofibroma should be distinguished from other benign spindle cell tumors as leiomyoma, fibrous histiocytoma, fibromatosis, and nodular fasciitis. In addition, some myofibromas can be multifocal (myofibromatosis) and present worrisome features as local destruction, mitotic activity, and subendothelial growth, mimicking vascular invasion. Awareness of these microscopic features can avoid the confusion with smooth muscle, fibroblastic, or myofibroblastic sarcomas [3].

The definitive treatment for a solitary myofibroma is conservative surgical excision, as myofibromas may regress spontaneously [4]. If tumor is extensive, morbidity will be decreased by conservative resection and clinical monitoring for recurrence in comparison to a more extensive resection to achieve negative margins. When the myofibroma is biopsy-positive and immunohistochemistry-positive, reported recurrence rates are of 0% with conservative resection [2].

In conclusion, myofibroma is a rare benign tumor of the head and neck which must be considered to prevent inappropriate and/or overaggressive treatment. The vast majority of tumors will not recur with complete, conservative resection, even with positive margins, although close clinical follow-up is essential to diagnose recurrence.
